# Supplementary material for: Laboratory Diagnostic of Acute Kidney Injury and Its Progression: Risk of Underdiagnosis in Female and Elderly Patients
Source: J Clin Med. 2023 Jan 30;12(3):1092. doi: 10.3390/jcm12031092 (PMC9917506; doi:10.3390/jcm12031092)
Supplement: Supplementary file 1 [file jcm-12-01092-s001.zip › jcm-2051813-supplementary.pdf]

Supplement

Text S1. Recalculation of Creatinine

Estimated glomerular filtration rate (eGFR, ml/min/1.73m²) values at ULMC are calculated on the basis of the CKI-EPI equations [17] (using SCr in µmol/L) for a predominantly non-black cohort thus:

Sex-specific knot mg/dL (µmol/L)	Equation
Females ≤ 0.7 (62)	eGFR = 144 × $(SCr/0.7)^{-0.329}$ × $(0.993)^{Age}$
Females > 0.7 (62)	eGFR = 144 × $(SCr/0.7)^{-1.209}$ × $(0.993)^{Age}$
Males ≤ 0.9 (80)	eGFR = 141 × $(SCr/0.9)^{-0.411}$ × $(0.993)^{Age}$
Males > 0.9 (80)	eGFR = 141 × $(SCr/0.9)^{-1.209}$ × $(0.993)^{Age}$

For the reverse action, the inference of serum creatinine (SCr) from eGFR, we rearranged the equations to the following while retaining the original grouping below and at/above the sex-specific knot, respectively. The factors 0.9 (instead of 0.7), 141 (instead of 144), and -0.411 (instead of -0.329) are needed to transform a "female" SCr into "male" SCr via the original eGFR. The recalculation of males into younger individuals was achieved by setting the variable Age to 30.

Below/at sex-specific knot	SCr = e ^ (ln (eGFR/141/0.993 ^ Age) / (-0.411)) * 0.9 * 88.42
above sex-specific knot	SCr = e ^ (ln (eGFR/141/0.993 ^ Age) / (-1.209)) * 0.9 * 88.42

Table S1. Comparison of progressive and non-progressive cases at first AKI detection during hospitalization in females and males. Variables are given as medians [interquartile range] or percentages. Significant *p*-Values (< 0.05) highlighted as bold.

	Female			Male		
	**Progressive AKI**	**Non-progressive AKI**	***p*-Value**	**Progressive AKI**	**Non-progressive AKI**	***p*-Value**
Incidence proportion, n, %	554, 22.0	2514, 78.0		904, 25.0	3620, 75.0	
**Basic patient characteristics**						
Age (years)	70.7 [59.0–79.5]	72.4 [60.7–81.1]	**0.013**	66.0 [57.0–75.9]	68.0 [58.2–78.1]	**0.003**
Total length of hospitalization (days)	24.7 [14.2–43.1]	15.1 [8.3–27.1]	**< 0.001**	26.3 [14.9–42.6]	16.0 [8.2–28.3]	**< 0.001**
First eGFR	58.0 [36.1–85.1]	60.2 [36.0–85.5]	0.696	61.9 [37.7–88.1]	63.7 [39.7–88.2]	0.371
Last eGFR	44.9 [25.2–74.4]	54.9 [34.4–83.6]	**< 0.001**	58.1 [38.1–85.7]	46.0 [25.7–75.7]	**< 0.001**
Time to first AKI during hospitalization (days)	4.9 [1.9–11.9]	4.6 [1.9–10.6]	0.242	4.5 [1.8–9.4]	4.5 [1.9–10.0]	0.336
In-hospital mortality	45.5	16.8	**< 0.001**	45.0	17.5	**< 0.001**
**Comorbidities**						
I10.- Hypertension	47.7	48.3	0.823	46.9	46.2	0.750
E11.- Diabetes mellitus	31.4	31.9	0.847	35.2	34.2	0.607
E86.- Exsiccosis	6.0	4.5	0.176	3.4	4.6	0.143
R57.- Shock	37.9	12.6	**< 0.001**	44.3	16.8	**< 0.001**
I25.- Coronary heart disease	11.2	14.0	0.097	23.5	23.3	0.980
I21.- Myocardial infarction	3.4	3.2	0.869	6.2	4.5	**0.046**
I50.- Cardiac insufficiency	33.9	27.8	**0.004**	34.6	26.4	**< 0.001**
A41.- Sepsis	38.8	15.6	**< 0.001**	41.9	20.7	**< 0.001**
K74.- Liver cirrhosis	8.7	4.3	**< 0.001**	9.0	4.1	**< 0.001**

Table S2. Comparison of age groups with regard to AKI stages and sex. Based on common AKI cases for females and females as male.

Age group		First AKI stage during hospitalization, %, (*n*)			Maximum AKI stage during hospitalization, %, (*n*)		
		**AKIN1**	**AKIN2**	**AKIN3**	**AKIN1**	**AKIN2**	**AKIN3**
[18–41)	Female	78.2 (140)	15.1 (27)	**6.7 (12)**	62.6 (112)	20.1 (36)	**17.3 (31)**
	Female as male	74.3 (133)	14.0 (25)	**11.7 (21)**	59.8 (107)	13.4 (24)	**26.8 (48)**
[41–61)	Female	81.3 (443)	12.1 (66)	**6.6 (36)**	61.3 (334)	22.6 (123)	**16.1 (88)**
	Female as male	83.1 (453)	10.8 (59)	**6.1 (33)**	62.2 (339)	20.7 (113)	**17.1 (93)**
[61–81)	Female	82.8 (1207)	11.5 (167)	**5.7 (83)**	65.5 (955)	20.9 (305)	**13.5 (197)**
	Female as male	83.1 (1211)	10.3 (150)	**6.7 (97)**	65.4 (953)	18.1 (264)	**16.5 (241)**
[81–max]	Female	86.2 (631)	10.5 (77)	**3.3 (24)**	71.7 (525)	20.1 (147)	**8.2 (60)**
	Female as male	85.1 (622)	9.4 (69)	**5.5 (40)**	69.4 (507)	17.1 (125)	**13.5 (99)**

Table S3. Comparison of age groups regarding AKI stages and sex. Based on common AKI cases for males and males as young.

Age group		First AKI stage during hospitalization, %, (*n*)			Maximum AKI stage during hospitalization, %, (*n*)		
		**AKIN1**	**AKIN2**	**AKIN3**	**AKIN1**	**AKIN2**	**AKIN3**
[18–41)	Male	74.2 (187)	17.9 (45)	**7.9 (20)**	57.5 (145)	23.8 (60)	**18.7 (47)**
	Male as young	76.6 (193)	15.1 (38)	**8.3 (21)**	59.5 (150)	20.2 (51)	**20.2 (51)**
[41–61)	Male	79.2 (795)	12.6 (127)	**8.2 (82)**	56.1 (563)	21.9 (220)	**22.0 (221)**
	Male as young	83.7 (840)	8.5 (59)	**7.9 (79)**	62.1 (623)	17.1 (172)	**20.8 (209)**
[61–81)	Male	82.7 (1827)	10.5 (232)	**6.8 (150)**	64.1 (1417)	18.4 (407)	**17.4 (385)**
	Male as young	83.7 (1848)	5.6 (123)	**10.8 (238)**	66.8 (1476)	10.7 (236)	**22.5 (497)**
[81–max]	Male	87.0 (578)	7.5 (50)	**5.4 (36)**	70.0 (465)	15.5 (103)	**14.5 (96)**
	Male as young	82.1 (545)	2.7 (18)	**15.2 (101)**	64.5 (428)	7.8 (52)	**27.7 (184)**
